# Cognitive Effects of Esketamine in Treatment-Resistant Depression: A Systematic Review

**DOI:** 10.1097/JCP.0000000000002165

**Published:** 2026-03-24

**Authors:** Riccardo Guglielmo, Emma Laura Facchinetti, Daniele Cioci, Miriam Olivola, Beatriz Pereira da Silva, Alessandra Mazzocchi, Bernardo Dell’Osso, Mario Amore, Gianluca Serafini

**Affiliations:** 1 Department of Neuroscience, Rehabilitation, Ophtalmology, Genetics, Maternal and Child Health, University of Genoa; 2 Psychiatric Unit, IRCCS Ospedale Policlinico San Martino, Genoa; 3 Department of Brain and Behavioural Sciences, University of Pavia, Pavia; 4 Dipartimento di Salute Mentale e Delle Dipendenze, ASST Fatebenefratelli-Sacco; 5 Department of Mental Health, Department of Biomedical and Clinical Sciences “Luigi Sacco”, University of Milan; 6 “Aldo Ravelli” Center for Nanotechnology and Neurostimulation, University of Milan, Milan, Italy; 7 Department of Psychiatry and Behavioral Sciences, Stanford University, Stanford, CA

**Keywords:** esketamine, treatment-resistant depression, cognition, memory, executive functions

## Abstract

**Background:**

Treatment-resistant depression affects up to one third of patients with major depressive disorder and is frequently accompanied by persistent cognitive impairments that significantly hinder functional recovery. Emerging evidence suggests that sub-anesthetic esketamine does not appear to be associated with cognitive deterioration and may be associated with procognitive effects; however, no systematic review has specifically synthesized cognitive outcomes in this population.

**Methods:**

A systematic search was conducted. Eligible studies included adults aged 18 to 80 years receiving intranasal or intravenous esketamine, with cognitive assessments performed at least 1 month after treatment initiation. Six studies met the inclusion criteria. Cognitive outcomes were grouped into attention/processing speed, memory, and executive functions.

**Results:**

Across studies, available evidence suggests that esketamine does not appear to be associated with cognitive deterioration in any assessed domain. Improvements in attention and processing speed were the most frequent and robust findings, observed in both RCT-derived datasets and naturalistic longitudinal studies. Memory remained stable in short-term designs but improved in studies with extended follow-up. Executive functions showed gains primarily among participants with baseline impairments and in long-term assessments. All remaining evaluations indicated cognitive stability.

**Conclusions:**

Current evidence, albeit limited, suggests that esketamine appears to be cognitively safe in adults with treatment-resistant depression and may confer selective improvements, particularly in attention and processing speed, with more variable benefits in memory and executive functioning over sustained treatment. These effects may support functional recovery and complement esketamine’s antidepressant action. Further research using harmonized cognitive batteries, larger samples, and longer follow-up periods is needed to better characterize cognitive trajectories and their relevance for personalized treatment strategies.

Depression is a highly prevalent and disabling psychiatric disorder, characterized by disturbances across emotional, neurovegetative, and neurocognitive domains. Current projections from the World Health Organization indicate that it will become the leading global cause of disease burden by 2030.^1^ Cognitive dysfunction is a well-recognized and clinically relevant component of major depressive disorder (MDD), affecting ~40% of patients across domains such as attention, memory, verbal fluency, and executive functioning.^2–4^ These impairments often persist even after the remission of mood symptoms and are associated with longer illness duration, increased recurrence risk, and poorer psychosocial and occupational functioning.^5^ Accumulating evidence further suggests that cognitive deficits may follow a progressive trajectory, with greater illness chronicity and recurrent depressive episodes linked to worsening performance in processing speed, attention, and working memory.^6^ Treatment-resistant depression (TRD), typically defined as failure to respond to at least 2 adequate antidepressant trials,^7^ affects roughly one third of patients with MDD and is associated with markedly worse clinical and functional outcomes. Individuals with TRD exhibit higher rates of hospitalization, suicidality, relapse, and medical comorbidities, along with substantial impairments in quality of life and social functioning.^8^ Cognitive dysfunction constitutes a major contributor to this burden. Compared with patients experiencing a first depressive episode, those with TRD display more pronounced deficits in executive functions and verbal working memory, as well as subtle impairments across additional domains.^9,10^ Multiple mechanisms have been proposed to explain these deficits, including illness progression, cumulative pharmacological exposure, and vulnerability factors that may themselves predispose to treatment resistance.^11^ In addition, emerging evidence suggests that cumulative antidepressant nonresponse, reflecting illness severity and treatment refractoriness, may predict poorer cognitive functioning and reduced responsiveness to subsequent interventions, including esketamine, highlighting the importance of integrating cognitive status and treatment history into personalized therapeutic planning.^12^ Pharmacological management of TRD remains challenging. Despite their widespread use, conventional antidepressants such as SSRIs, SNRIs, and tricyclics have not demonstrated consistent cognitive benefits in TRD, with vortioxetine representing the only agent with documented procognitive efficacy in MDD, though this has not been specifically confirmed in TRD populations.^13,14^ Esketamine, the S-enantiomer of ketamine and an NMDA receptor antagonist, has emerged in recent years as an important therapeutic option for TRD.^15^ The intranasal formulation (esketamine nasal spray) is approved by major regulatory agencies, including the US Food and Drug Administration and the European Medicines Agency, for use in adults with TRD and in adults with MDD with acute suicidal ideation or behavior, in conjunction with an oral antidepressant or alone, and is authorized for clinical use in multiple countries worldwide. Both intravenous and intranasal formulations, when combined with oral antidepressants, produce rapid and robust antidepressant effects with acceptable tolerability. Although its long-term safety profile is still under investigation,^16^ an increasing body of evidence suggests that esketamine is not cognitively impairing and may even exert beneficial effects on specific cognitive domains in patients with TRD.^10,17^ Although ketamine and esketamine share a common glutamatergic mechanism of action, the 2 compounds differ substantially in stereochemistry, potency, and pharmacokinetic properties. Esketamine (the S-enantiomer) exhibits greater affinity for the NMDA receptor and higher anesthetic potency compared with the R-enantiomer and racemic formulations, as demonstrated in classical pharmacological studies and subsequent mechanistic reviews.^18,19^ Furthermore, esketamine’s absorption profile, metabolism, and clinical monitoring requirements differ from those of intravenous racemic ketamine, reflecting its distinct pharmacokinetic behavior and regulatory evaluation. Importantly, esketamine is the only glutamatergic agent formally approved for the treatment of TRD, with standardized intranasal dosing protocols and mandated postadministration monitoring. In contrast, research on ketamine encompasses heterogeneous dosing schedules, administration routes, clinical populations, and treatment settings, which complicates the interpretation of cognitive outcomes and limits the comparability of findings across studies. For these reasons, and to ensure methodological coherence, the present review focuses exclusively on esketamine, allowing the evaluation of cognitive effects within a unified therapeutic and regulatory framework. From a neurobiological standpoint, glutamatergic modulation by esketamine is thought to enhance synaptic plasticity within fronto-limbic circuits, potentially normalizing network dynamics that support attention, working memory, and executive control.^6,14^ Beyond esketamine-specific findings, several controlled experimental studies on ketamine have reported acute improvements in episodic memory, visuospatial processing, and attentional performance, supporting the hypothesis that glutamatergic modulation may enhance cognition when administered at precise sub-anesthetic levels.^20^ Moreover, repeated sub-anesthetic ketamine infusions have not been associated with cognitive deterioration in either adult or adolescent populations.^21^ Collectively, these findings substantiate the rationale for a systematic evaluation of esketamine’s cognitive effects in TRD. In parallel, the personalization of treatment for TRD has become a central objective in contemporary psychopharmacology. Predictive markers of esketamine response, including clinical features, temperament dimensions, and indicators of cumulative treatment resistance, have started to emerge,^12,22^ yet the extent to which cognitive profiles and cognitive trajectories may inform patient selection and long-term treatment planning remains largely unexplored. Clarifying whether esketamine merely preserves cognitive functioning or can contribute to cognitive recovery is therefore crucial for integrating this agent into individualized care pathways for TRD. Given the central role of cognition in functional recovery and the emerging emphasis on personalized treatment approaches in TRD, a systematic synthesis of the available evidence is warranted. The present review aims to systematically evaluate the medium-term to long-term cognitive effects of esketamine in adults with TRD, with the goal of clarifying its cognitive safety profile, identifying domains most likely to improve, and informing clinical decision-making within personalized care frameworks.

## MATERIALS AND METHODS

### Protocol Registration

This systematic review was registered in the International Prospective Register of Systematic Reviews (PROSPERO) under the registration number CRD420251243697. The protocol was developed following the Preferred Reporting Items for Systematic Reviews and Meta-Analyses guidelines.^23^ As this review synthesizes data from previously published studies, no additional ethics approval was required, given that ethical approval had already been obtained in all original investigations.

### Eligibility Criteria

We included studies that examined the effects of intranasal or intravenous esketamine on cognitive functioning in adults (18 to 80 years) with TRD, diagnosed according to The Diagnostic and Statistical Manual of Mental Disorders, 5th edition, (DSM-5)^24^ or ICD-11 criteria. Eligible designs comprised randomized controlled trials, open-label longitudinal studies, and observational follow-up studies using standardized neuropsychological measures. To ensure that cognitive outcomes reflected postacute cognitive performance, we included assessments conducted outside the immediate postdose window (ie, at least 24 hours after administration), thereby minimizing the influence of transient dissociative or perceptual effects. Studies varied in follow-up duration: some evaluated cognition in the early subacute phase (eg, within the first 10 to 14 days of treatment), whereas others assessed sustained effects at 4 to 12 weeks or during long-term maintenance. We excluded studies that reported only within-session or immediate postsession testing, studies without standardized cognitive measures, studies involving racemic ketamine without esketamine-specific analysis, animal or in vitro studies, samples without formal DSM/ICD diagnoses, and articles written in languages other than English, Italian, French, Spanish, or Portuguese.

### Information Sources and Search Strategy

A systematic literature search was conducted in PubMed, PsycINFO, and Scopus, with the final search completed in February 2025. No publication date limits were applied. The search strategy combined controlled vocabulary and free-text terms referring to esketamine and cognitive functioning, and was adapted to the indexing structure of each database. The complete search strategy for each database is systematically detailed in the Supplemental Digital Content, http://links.lww.com/JCP/B25. Reference lists of all included studies were also reviewed to identify additional eligible publications.

### Selection Process

For the selection of articles, the tool Rayyan.ai^25^ was used by 2 examiners who operated blindly. Initially, with the help of the automatic tool provided by the platform, duplicates were screened out. Subsequently, the 2 reviewers independently selected the articles, first evaluating titles and abstracts, and then examining the full text of potentially eligible studies. Discrepancies were resolved through discussion and, if necessary, with the involvement of a third reviewer.

### Data Collection Process

A standardized data extraction form was developed and piloted to systematically collect relevant information from each included study. The collected information included author, year, study design, population characteristics, esketamine administration protocol, neurocognitive assessment tools, and main outcomes. Extraction was performed independently by 2 reviewers, and disagreements were resolved by consensus.

### Data Items

The primary variable of interest was the presence and direction of change in neurocognitive performance associated with intranasal or intravenous esketamine treatment. The main outcomes were the variations in cognitive performance, grouped into the 3 domains of memory, attention and executive functions, and assessed through validated neuropsychological tests at different time points (baseline, short-term and long-term follow-up). The results were summarized narratively.

### Risk of Bias and Quality Assessment

The methodological quality of the included studies was assessed at the individual study level using the NIH Quality Assessment Tool, applying the checklist appropriate to each study design (before-after studies without control, case series, or systematic review). Two reviewers independently performed the assessments, resolving disagreements through consensus. Funding sources and authors’ declared conflicts of interest were also reviewed to contextualize potential biases. The NIH tool evaluates multiple domains of methodological rigor, including the clarity of the research question, definition and selection of study participants, comparability of groups when applicable, ascertainment of exposure, blinding of outcome assessment, sample size justification, and the handling of potential confounding variables. Across the 6 included studies, none were rated as poor.

Two studies were rated as good, reflecting clearly defined research aims, appropriate eligibility criteria, standardized neurocognitive assessments, and adequate control of methodological confounders. The remaining 4 studies were rated as Fair, primarily due to limitations inherent to their design. Common factors contributing to Fair ratings included lack of blinding of assessors, incomplete adjustment for relevant confounders (such as concomitant psychotropic medications, benzodiazepine use, or illness chronicity), insufficient reporting of attrition, and the absence of sample size justification. In before-after designs, the lack of control groups also limited causal inference regarding cognitive changes. Despite these limitations, all studies employed validated neuropsychological instruments and consistent pre-post assessment procedures, supporting the internal validity of the observed cognitive outcomes. A detailed study level summary of NIH quality ratings is presented in Tables S1–S3, Supplemental Digital Content, http://links.lww.com/JCP/B25.

### Synthesis Methods

Given that this review aimed to qualitatively summarize the available evidence, a narrative synthesis approach was applied. The cognitive outcomes reported in the included studies were grouped into 3 domains, attention/processing speed, memory, and executive functions, based on shared conceptual frameworks and the structure of the neuropsychological instruments used. The findings were then synthesized descriptively, highlighting the direction and clinical relevance of changes observed over time within each domain. The synthesis was conducted independently by 2 reviewers, and any interpretive discrepancies were resolved through discussion. To account for differences in timing, outcomes were interpreted according to whether they reflected subacute (eg, within 2 weeks) or sustained cognitive effects (≥ 4  weeks).

### Data Availability

All data included in this review are derived from previously published studies. Extracted numerical data (eg, means, effect sizes, SDs, and CIs) are available directly within the original publications listed in Table 1.

**TABLE 1:** Main Findings

Study	Study Design	Study Population	Duration	Form of Administration	Time of Evaluation	Medication	Neurocognitive Assessment Tools	Neurocognitive Functions at Baseline (Mean)	Main Outcomes
Yang et al^17^	Open-label, longitudinal trial	N (132), age (y) 25.61 ± 14.26, sex M=49 (37.12%); *F*=83 (62.88%)	11 d	6 sub-anesthetic ESK infusions were administered in participants over 11 d (days 1, 3, 5, 7, 9, and 11) at a dose of 0.40 mg/kg.	Baseline; 24 h after 6th IV infusion (day 12)	FLUeq(mg/d) 65.34 ± 33.00 (129); CPZeq(mg/d) 206.51 ± 223.31 (24); DPeq(mg/d) 11.36 ± 9.25 (72)	RBANS	RBANS 203.78 ± 29.29; immediate memory 36.03 ± 9.16; delayed memory 45.86 ± 7.07; visuospatial/constructional 34.86 ± 3.52; language 26.81 ± 4.78; attention 60.06 ± 13.04; Plasma NGF concentrations (ng/mL) 226.13 ± 61.73.	After 6 esketamine infusions, patients exhibited significant improvement in global cognition and multiple RBANS subdomains. RBANS total score increased to 226.13 ± 29.42 (*t*=13.838, *P*<0.001). Memory composite scores (immediate + delayed) improved substantially to 96.84 ± 14.68 (*t*=17.512, *P*<0.001), with significant gains also observed in language and attention, while visuospatial/constructional abilities remained unchanged. NGF plasma levels increased from 226.13 to 384.37 ng/mL, paralleling cognitive improvements. Baseline memory performance negatively correlated with both changes in MADRS (*r*=–0.226, *P*=0.024) and NGF (*r*=–0.352, *P*<0.001), indicating that poorer baseline memory predicted greater antidepressant and neurotrophic response. No cognitive deterioration was reported.
Morrison et al^25^	Pooled analysis of 4 phase 3, double-blind, multicenter studies	N (884), age (y) 51.4, sex *F*=661; Comparison group: PBO + OAD (432)	6 mo	ESK nasal spray or PBO plus a newly initiated daily OAD was administered twice weekly for 4 weeks with a fixed ESK dose or flexible ESK dose (28/56/84 mg). In the 12-wk optimization phase, ESK dosing frequency was reduced to once weekly for 4 wk, then individualized based on the severity of depressive symptoms.	Baseline; day 28; 2 wk follow-up	1 from among 4 ADs: escitalopram, duloxetine, sertraline, or venlafaxine extended release; OAD was administered daily in an OL manner and was to be continued throughout the study.	Cogstate computerized test battery, 5 domains: IDN, DET, OCL, GMLT and ONB; HVLT-R 2 domains: delayed recall and total recall	Seven domains of cognition were measured using 5 tests from the Cogstate computerized test battery (IDN, DET, OCL, GML and ONB) and 2 scores from HVLT-R (delayed recall and Total recall).	Esketamine + OAD produced significantly greater improvement than placebo + OAD in: •Identification Speed (IDN): primary marker of processing speed (CI 95% ≠ 0) • HVLT-R Delayed and Total Recall: indicating enhanced verbal learning and memory retention. Both treatment arms improved on DET, OCL, GMLT, and ONB, with no cognitive deterioration observed in either group. Findings indicate selective procognitive advantages of esketamine in processing speed and verbal memory during the induction phase.
Pepe et al^26^	Open-label, longitudinal, case series	N (8), age 52.9(y), sex *F*=3, M=5	3 mo	ESK nasal spray was administered twice a week at a dosage of 56 mg for the first month and subsequently increased to 84 mg with once-a-week administration over the following 8 wk.	Baseline; 4 wk; 8 wk; 12 wk	Atypical antipsychotics (3), benzodiazepines (3), different classes of antidepressants (8), mood stabilizers (2), not modified throughout the observation period	DSST; TMT-B; PDQ-D5	DSST 36.4; TMT-B 149; PDQ-D5 10.9.	Progressive improvement across all measures: DSST increased from 36.4 → 48.9; TMT-B improved from 149 → 72.9 at 12 wk; PDQ-D5 decreased from 10.9 → 5 over 12 wk. No statistical testing reported, but descriptive trends indicate clinically meaningful gains in processing speed, executive functioning, and subjective cognitive efficiency.
Wais et al^27^	Phase 3, open-label, multicenter, longitudinal	N (802), age (y) 52.2 ± 13.69, sex M=300 (48.4%) *F*=502 (62.6%)	Median exposure 22.9 wk	ESK nasal spray at a dosage of 56 mg (IND: 45.8%; OP/MAINT: 45.6%) / 84 mg (IND: 48.8%; OP/MAINT: 50.2%) / 28 mg (IND: 5.3%; OP/MAINT: 4.0%). During the OP/MAINT phase, 24.0% of patients received weekly dosing, 38.1% were maintained on every-other-week dosing, and 37.8% switched more than once between weekly and every-other-week dosing	Baseline; Day 28; week 20; week 32; week 44	Duloxetine 251 (31.3%) Escitalopram 237 (29.6%) Sertraline 157 (19.6%) Venlafaxine XR 156 (19.5%)	Cogstate computerized test Battery and HVLT-R	Cogstate computerized test Battery and HVLT-R	Performance across all Cogstate and HVLT-R domains remained stable or improved through week 44, including reaction time, visual and verbal learning, memory, working memory, and executive function. No cognitive deterioration was observed. Findings indicate that long-term esketamine treatment is cognitively safe, with evidence of gradual improvement in several domains over sustained exposure.
Sobreiro et al^28^	Longitudinal, open-label preliminary report	N (8), age (y) 49.62, *F*=4 (50%)	6 mo	Self-administered ESK nasal spray under a psychiatrist’s supervision (28/56/84 mg)	T0 (baseline); T3 (~3 mo); T6 (~6 mo)	Patients and their treating psychiatrist were instructed to maintain the ongoing treatment regimen, including the prescribed medications, both during and following the cessation of esketamine therapy.	FAS; Animal Fluency test was used to assess semantic fluency; Backward Digit Span test assesses working memory; Stroop Test, Victoria Version used to assess inhibitory control; WCST assesses the ability to develop and maintain an appropriate problem-solving strategy across changing stimulus conditions; TMT-A, TMT-B assesses attention, visual search, speed of processing, sequencing and shifting; WAIS-III-R Flexibility and executive functioning; WMS Logical Memory I-II; RAVLT, 5 domains	FAS: 37.9 (10.0); Animal Fluency test: 17.0 (4.2); Backward Digit Span test: 5.8 (1.7); Stroop Color: 29.4 (10.5);. WCST, Total Correct: 103.9 (26.4). TMT: TMT-A: 39.5 (16.6); TMT-B: 72.8 (33.6). WAIS-III-R: Block 24.0 (13.1), Vocabulary 47.9 (15.4); WMS Logical Memory I: 40.5 (22.4), Logical Memory II: 31.7 (24.3). RAVLT Correct 52.9 (15.5), immediate memory span 4.8 (2.7), 30 min. recall 7.9 (4.0), delayed recall 9.2 (2.9), recognition 13.1 (2.1)	Moderate improvements emerged primarily in attention, processing speed, and working memory, with significant gains on: - TMT-A (*P*<0.05 at T3 and T6): 32.5(11.4) at T3, 29.4(7.9) at T6; - Digit Span Backward (*P*<0.05 between T3 and T6): 6.0(1.9) at T3, 7.6(2.2) at T6; - RAVLT (Correct and Immediate scores improved at T3; *P*<0.05); Correct: 64.8(13.8) at T3, 68.8(10.2) at T6; Immediate: 5.5(1.6) at T3, 6.8(1.8) at T6. - WMS Logical Memory I-II (*P*<0.05 at T3) LM-I: 53.0(24.5) at T3, 54.0(25.1) at T6. LM-II: 49.8(26.3) at T3, 45.3(29.2) at T6. - Stroop Color (*P*<0.05 at T3); 26.1(6.7) at T3, 25.8(7.9) at T6. Additional gains at T6 included: - WAIS-III Vocabulary (*P*<0.05) - WCST Total Correct (*P*<0.05) Other measures showed stability, with no evidence of cognitive decline across the 6-mo follow-up. Overall, findings suggest selective improvement in attention, working memory, and episodic memory, with preserved performance in other domains.
Pepe et al^29^	Open-label, longitudinal	25 (56% males, 55.1 10.9 y)	3 mo	ESK nasal spray (mean dose: 78.4 11.43 mg)	Baseline; 4 wk; 8 wk; 12 wk.	SSRIs 17 (68) SNRIs 9 (36) other ADs 10 (40) Mood stabilizers/ anticonvulsants 14 (56) Antipsychotics 11(44) Sedative hypnotics/anxiolytics 12 (48)	DSST; PDQ-D5	DSST 39.1 (13.2); PDQ-D5 9.45 (4.89)	DSST: Progressive improvement over 12 wk, with statistically significant gains at 8 wk and 12 wk (*P*=0.004; *P*=0.008), indicating enhanced processing speed and attentional efficiency; 4 wk: +2.29(1.78) *P*=0.205; 8 wk: +5.13(1.66); 12 wk: +4.86(1.74); PDQ-D5: Significant reductions in subjective cognitive difficulties at 8 and 12 wk 4 wk: 1.50 (0.82) *P*=0.072; 8 wk: 2.19(0.71); 12 wk: 2.15(0.87) No cognitive decline observed.

ADs, antidepressants; CPZeq, chlorpromazine equivalent milligrams; DB, double-blind; DET, Detection; DPeq, diazepam equivalent milligrams; DSST, Digit Symbol Substitution Test; ESK, esketamine; FAS, Verbal Fluency Test; FLUeq, fluoxetine equivalent milligrams; GML, Groton Maze Learning; HVLT-R, Hopkins Verbal Learning Test–Revised; HVLT-R_D, HVLT-R Delayed Recall; IDN, Identification; IND, induction; MADRS, Montgomery–Åsberg Depression Rating Scale; NGF, nerve growth factor; OAD, oral antidepressant; OCL, One-Card Learning; OL, open-label; ONB, One-Back Memory; p, statistical significance; PBO, placebo nasal spray; PDQ-D5, Perceived Deficits Questionnaire Depression-5 item; RAVLT, Rey Auditory Verbal Learning Test; RBANS, Repeatable Battery for the Assessment of Neuropsychological Status; SNRI, serotonin and norepinephrine reuptake inhibitor; SSRI, selective serotonin reuptake inhibitor; TMT, Trail Making Test; TMT-A, Trail Making Test-A; TMT-B, Trail Making Test-B; WAIS-III-R, Wechsler Adult Intelligence Scale-Third Edition Revised; WCST, Wisconsin Card Sorting Test; WMS LM-I, WMS Logical Memory I; WMS LM-II, WMS Logical Memory II; WMS, Wechsler Memory Scale; XR, extended release; y, year.

## RESULTS

### Study Selection

**FIGURE 1:**
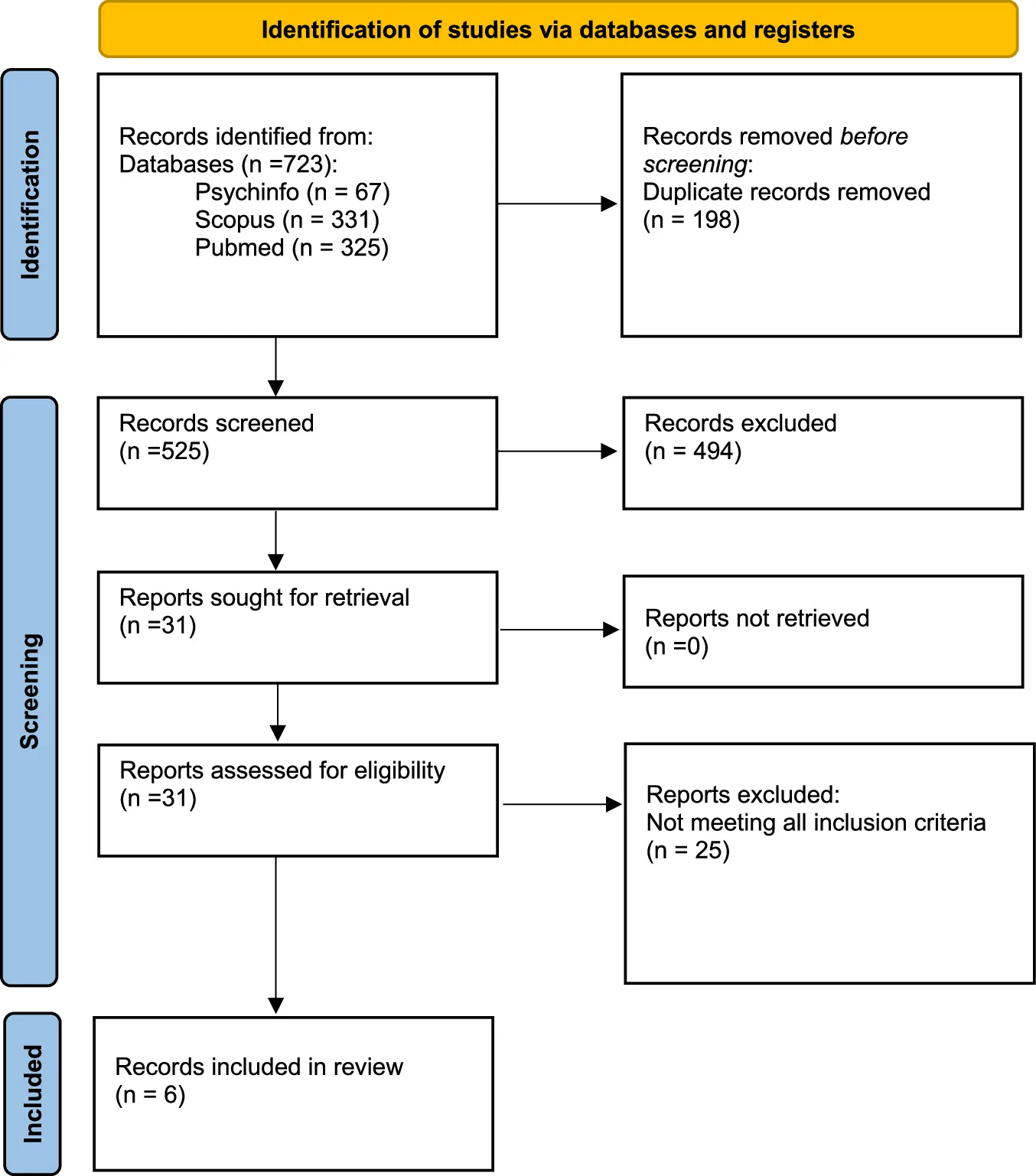
PRISMA flow diagram.

The systematic search conducted on 3 databases (PubMed, PsycINFO, and Scopus) returned a total of 723 articles. After the removal of 198 duplicates, 525 articles were screened. At this stage, based on the reading of the abstracts, 494 irrelevant articles were excluded. The remaining 31 articles were evaluated in full text. Of these, 25 were excluded because they did not meet the established inclusion criteria. At the end of the process, 6 studies were included in the review. The PRISMA flow diagram summarizing the selection process is presented in Figure 1.

### Study Characteristics

The main characteristics of the studies are summarized in Table 1. The studies were published between 2020 and 2025. The included studies comprised one pooled analysis of 4 phase 3 randomized controlled trials, one large open-label multicenter longitudinal extension study, one open-label intravenous esketamine trial, 1 small open-label long-term intranasal esketamine study, and 2 longitudinal clinical case series. Sample sizes ranged from 8 to 884 participants, and follow-up windows ranged from day 12 to 10 months. Cognitive outcomes were evaluated using standardized neuropsychological tests, including RBANS, Cogstate, DSST, TMT-A/B, RAVLT, Stroop, Digit Span, and Verbal Fluency. In all studies, cognitive assessments were performed at least 1 month after treatment initiation, minimizing the influence of acute dissociative effects. The most common intervention was the administration of intranasal esketamine at dosages of 28 mg (0.40 mg/kg), 56 mg, and 84 mg, generally on a weekly basis. Treatment regimens varied from 6 to over 20 administrations. Importantly, all assessments were performed outside the acute postdose dissociative window, with some capturing early subacute effects (≤2 weeks ) and others assessing sustained (4 to 12 weeks) or long-term (≥3 to 10 month) cognitive changes.

### Results of Syntheses

Three main cognitive domains have been identified for the synthesis of the results: attention/processing speed, memory, and executive functions. Table 2 summarizes the direction of the effects observed in each domain across different studies (positive, negative, or none). A conservative, rule-based classification strategy was applied. Outcomes were coded as “positive” only when at least one validated, performance-based neuropsychological measure within a given domain demonstrated a statistically significant or clearly reported clinically meaningful improvement at follow-up. Stable performance, defined as the absence of statistically significant change over time, was coded as “none”, while “negative” indicated statistically significant deterioration. When multiple subtests within the same cognitive domain yielded discordant results, domain-level classification was determined by the direction of the predominant and statistically supported effect, prioritizing objective performance-based measures over subjective reports. In controlled studies, classification was based on change relative to the control condition, such that outcomes were considered positive only when improvements exceeded those observed in the comparator group, thereby accounting for nonspecific effects such as practice effects or symptom-related fluctuations. Outcome classification was based exclusively on objective, performance-based neuropsychological tests; clinical impression, mood scales (eg, MADRS), or changes in depressive symptom severity were not used to define cognitive improvement. Using this conservative approach, esketamine showed the most consistent effects in the domain of attention and processing speed (positive in 4/6 studies). Memory outcomes were positive in studies with extended follow-up, while remaining stable in others (positive in 2/6). Executive functions improved primarily in longitudinal designs involving participants with baseline impairment (positive in 2/6). No study reported cognitive deterioration in any domain.

**TABLE 2:** Direction of Cognitive Change Across Included Studies

Cognitive Domain	Studies Assessing Domain (n)	Total Sample Size (N)	Positive	Negative	None
Attention/processing speed	6	1859	4	0	2
Memory	4	1826	2	0	2
Executive functions	4	1702	2	0	2

The table summarizes the direction of cognitive change reported across the included studies. “Positive” indicates statistically significant or clinically meaningful improvement in at least one validated cognitive measure within the specified domain. “None” indicates cognitive stability, defined as the absence of significant change over time. “Negative” reflects significant deterioration; no domain showed negative cognitive outcomes in the reviewed studies. Total sample size (N) reflects the cumulative number of participants across studies that assessed each cognitive domain; studies not evaluating a given domain were excluded from that domain’s counts.

### Attention and Processing Speed

Attention and processing speed showed consistent patterns of improvement across studies, although the strength of evidence varied by study design. In Yang et al,^17^ repeated intravenous esketamine infusions were associated with early subacute improvements in attentional performance at day 12, as measured by the RBANS Attention Index, indicating that cognitive changes may emerge within the first 2 weeks of treatment, independent of full symptomatic remission. In the large pooled RCT analysis,^27^ esketamine demonstrated greater improvement in processing speed and attentional performance compared to control treatment, as indexed by standardized Cogstate composite scores.^27^ In the phase 3 extension study, performance on attention and processing speed assessments remained stable or slightly improved across the maintenance period, with all evaluations conducted predose, indicating that improvements were not attributable to acute drug effects.^26^ Both case series reported meaningful improvements in processing speed.

In contrast, evidence from open-label designs should be interpreted more cautiously. In Pepe et al,^29^ performance on the Digit Symbol Substitution Test (DSST) showed a progressive and continuous improvement across the 12-week treatment course. Based on the individual patient-level values reported in the original table, the mean DSST score increased from 36.4 at baseline to 42.6 at 4 weeks, 44.0 at 8 weeks, and 48.9 at 12 weeks. However, given the absence of a control condition, these changes cannot be unequivocally attributed to esketamine and may reflect practice effects, nonspecific clinical improvement, or the natural course of treatment.

Similarly, in another open-label study,^28^ DSST performance increased over time, with a nonsignificant change at 1 month followed by statistically significant improvements at 2 and 3 months. While these findings suggest temporal improvement in processing speed, the lack of a comparator group precludes conclusions regarding superiority over treatment as usual or placebo.

### Memory

Memory performance across studies was generally characterized by stability or gradual improvement. In Yang et al,^17^ immediate and delayed memory improved significantly on RBANS at day 12, alongside increases in NGF levels, supporting a possible neuroplasticity-mediated mechanism. In Sobreiro et al,^30^ verbal memory and working memory improved over the 6-month intranasal treatment period, with effects becoming more pronounced at longer follow-up intervals. In the pooled phase 3 RCT dataset, memory performance was stable to mildly improved, with no evidence of cognitive decline.^27^ Likewise, in the large long-term extension study, memory assessed via the Cogstate One-Card Learning (OCL) test and, in a subset of participants, the HVLT-R, remained stable or showed small improvements, with all assessments conducted predose, indicating that findings reflected persistent cognitive functioning rather than transient postdose effects.^26^ Taken together, the available evidence suggests that esketamine does not impair memory in adults with TRD and may, in some cases, enhance memory performance, particularly over longer treatment durations.

### Executive Functions

Executive function outcomes showed a pattern of stability or gradual improvement, with heterogeneity reflecting differences in study design and baseline cognitive status. In Morrison et al,^27^ both treatment groups (esketamine + antidepressant vs. antidepressant + placebo) demonstrated similar improvements in composite neurocognitive performance, with no significant between-group differences, indicating that esketamine did not impair executive functioning, but did not confer an additional executive advantage relative to standard antidepressant therapy. In contrast, longitudinal studies suggested progressive improvement in executive control. In Pepe and colleagues, performance on TMT-B improved across 12 weeks, consistent with enhanced cognitive flexibility.^29^ Sobreiro et al,^30^ reported gains in working memory, inhibitory control, and set-shifting during 3 to 6 months of intranasal treatment. Importantly, in the large long-term extension study,^26^ performance on executive-linked tasks from the Cogstate battery, particularly the Groton Maze Learning Test (problem-solving and strategy learning) and the one-back task (working memory), remained stable or showed slight improvement, with assessments conducted pre-dose, confirming the absence of acute confounding and supporting the cognitive safety of sustained esketamine treatment. Across all studies, no deterioration in executive functioning was observed.

## DISCUSSION

This systematic review examined the effects of intranasal and intravenous esketamine on cognitive functioning in adults with TRD, across early subacute (≤ 2 weeks), sustained (4–12 weeks), and long-term (≥ 3–12 months) treatment windows. Three key findings emerged. First, no study reported cognitive deterioration in any domain. Second, the most consistent improvements were observed in attention and processing speed, with effects detectable as early as day 12 and consolidating across weeks to months. Third, memory and executive functions tended to remain stable, with improvements emerging primarily in studies with longer follow-up durations or in samples with greater baseline cognitive impairment. These findings are clinically meaningful. Cognitive impairment is a common and functionally disabling feature of major depression, and is especially pronounced in treatment-resistant cases, where attentional slowing, inefficient information processing, and working memory deficits persist even when mood symptoms partially improve. Such deficits are strongly associated with impaired occupational functioning, reduced social participation, and decreased likelihood of functional recovery. The observation that esketamine does not worsen cognition, and may in some cases improve cognitive functioning, therefore has direct relevance to patient-centered outcomes and long-term functioning.

Attention and processing speed demonstrated the clearest and most reproducible improvements. Early subacute gains, such as those observed in Yang et al,^17^ suggest that esketamine may exert direct effects on neural circuits supporting cognitive control, rather than improvements being solely secondary to mood change. Esketamine acutely increases glutamatergic transmission, enhances AMPA receptor throughput, and initiates mTOR-dependent synaptogenesis, promoting rapid strengthening of prefrontal cortical networks.^31–33^ Functional neuroimaging studies corroborate these mechanisms, showing increased functional connectivity within fronto-striatal and fronto-hippocampal circuits following ketamine administration.^34^ These circuit-level adaptations align with the progressive improvements observed over weeks to months in some studies,^28–30^ suggesting that attentional improvements may reflect both rapid synaptic modulation and longer-term circuit stabilization. In contrast, the pooled RCT findings showed improvements in both treatment arms, consistent with the contribution of symptom recovery and antidepressant co-administration.^27^ Taken together, these data indicate that esketamine appears cognitively safe and may confer selective benefit in attentional and processing domains in a subset of patients.

Memory performance was generally stable and improved during longer-term follow-up. The improvements in immediate and delayed recall observed in Yang et al^17^ and the sustained gains reported in Sobreiro et al^30^ suggest that enhanced memory performance may be tied to experience-dependent neural remodeling. Esketamine increases circulating BDNF and NGF, neurotrophic factors that promote hippocampal synaptic plasticity.^35^ That memory remained stable over prolonged treatment in Wajs et al,^26^ further supports the absence of cumulative cognitive toxicity, a central concern in maintenance therapies for TRD.

Executive function outcomes were more heterogeneous. Improvements were most evident in patients with lower baseline cognitive performance and during longer follow-up windows, as seen in Pepe et al^29^ and Sobreiro et al.^30^ This variability is consistent with models positing that executive control deficits in depression arise not only from synaptic dysfunction but also from disrupted inhibitory interneuron regulation within the prefrontal cortex. Ketamine has been shown to restore GABAergic transmission, reducing maladaptive cortical noise and facilitating more efficient top-down control.^36^ The absence of decline in any study provides convergent evidence for the cognitive safety of esketamine. Notably, the temporal pattern of cognitive change appeared consistent across domains: early modulation in attentional efficiency, followed by stabilization, and later reinforcement of working memory and executive processes in longer follow-up windows. This gradient is coherent with models of progressive synaptic strengthening and network reorganization during sustained treatment.

Against this background, a theoretical concern relates to the long-term cognitive consequences of repeated NMDA receptor modulation. Although NMDA receptors play a central role in learning and synaptic plasticity, available longitudinal clinical data do not currently suggest cumulative cognitive deterioration associated with esketamine treatment, while longer follow-up remains necessary to fully exclude delayed effects.

In parallel, preclinical studies have suggested differential effects of ketamine enantiomers on cognitive outcomes. In animal models of phencyclidine-induced cognitive impairment, repeated intermittent administration of (R)-ketamine, but not (S)-ketamine, ameliorated recognition memory deficits and restored prefrontal and hippocampal neurotrophic signaling.^37^ These findings have raised important hypotheses regarding enantiomer-specific mechanisms of cognitive modulation. However, their translational relevance to TRD remains uncertain. Preclinical paradigms rely on acute pharmacological models that do not fully capture the clinical complexity of TRD, including illness chronicity, comorbidities, and concomitant pharmacotherapy. Within this context, the present findings indicate that, despite enantiomer-specific differences observed in experimental models, available human evidence supports the cognitive safety of esketamine in clinical practice.

Taken together, the evidence suggests that esketamine is not cognitively impairing and may improve cognitive domains that are especially relevant for functional recovery, particularly attention, processing speed, and working memory. Importantly, improvements in cognitive functioning may enhance patients’ capacity to engage with psychotherapy, initiate behavioral activation, and reintegrate into occupational and social roles. This suggests that the therapeutic value of esketamine may extend beyond mood reduction to include support for cognitive recovery, a treatment target increasingly recognized as central to remission.

**FIGURE 2:**
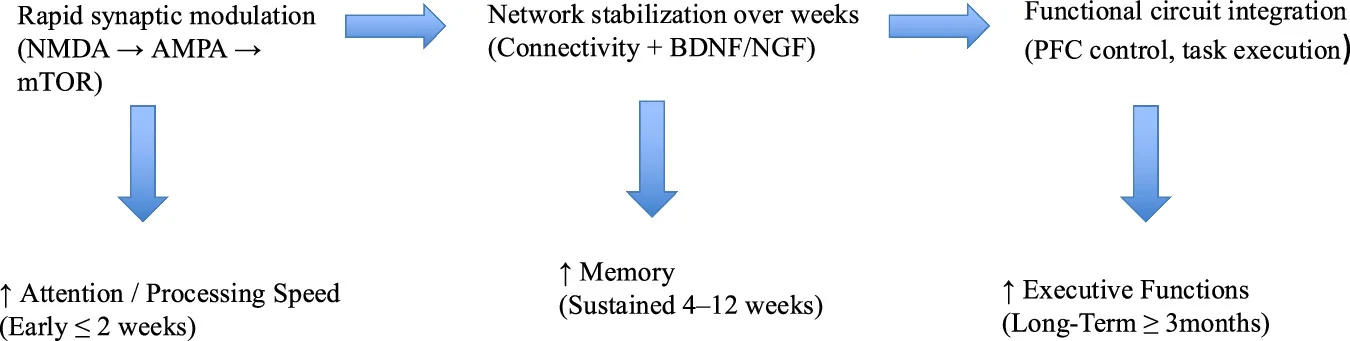
Conceptual model of cognitive change associated with esketamine treatment. Rapid synaptic modulation via NMDA antagonism, AMPA throughput, and mTOR activation is associated with early improvements in attention and processing speed. With continued treatment, network-level stabilization supported by neurotrophic signaling (BDNF/NGF) is associated with emergent gains in memory. Over longer durations, functional integration of prefrontal control networks may facilitate improvements in executive functioning, particularly in individuals with baseline executive impairment. The model illustrates a temporal progression rather than discrete or isolated effects.

Overall, the pattern observed across studies suggests that cognitive changes unfold gradually rather than uniformly, with attentional efficiency improving first and executive functioning emerging more slowly over ongoing treatment. Figure 2 provides a concise conceptual representation of this proposed trajectory, illustrating how different cognitive domains may recover at different rates during esketamine therapy.

### Clinical Implications

These findings have direct relevance for clinical practice. Cognitive impairment is increasingly recognized as a core dimension of depression, particularly in treatment-resistant forms, where attentional slowing, psychomotor inefficiency, and working memory deficits often persist beyond mood symptom improvement and contribute to incomplete functional recovery. Such deficits are among the strongest predictors of reduced work performance, social withdrawal, and long-term disability, and patients themselves frequently report cognitive symptoms as a primary determinant of quality of life and daily functioning.^38^ Therefore, interventions that are cognitively neutral or supportive, rather than cognitively suppressive, are of substantial clinical importance.^39^ The finding that esketamine does not impair cognition even with continued administration is particularly relevant in this context, given that many pharmacological treatments used in recurrent or chronic depression (eg, benzodiazepines, sedating antidepressants, anticholinergic adjuncts) may negatively influence processing speed, attention, and memory.^40^

In contrast, the pattern observed here, early improvements in attention and processing speed, followed by stability or enhancement in memory and executive functions over time, suggests that esketamine may contribute to a restoration of cognitive efficiency, particularly in patients presenting with a profile of cognitive fatigue, slowed information processing, or impaired task initiation. These cognitive changes may also have important downstream functional effects. Improvements in processing speed and attentional regulation can enhance a patient’s ability to engage in psychotherapeutic work, sustain behavioral activation strategies, and re-establish routines. Likewise, the stabilization of working memory and executive functions over longer treatment intervals may facilitate planning, goal-directed behavior, and role functioning, which are central to recovery beyond symptom reduction. The observation that cognitive improvements may occur prior to or in parallel with mood improvement suggests that targeting cognitive functioning early in the treatment trajectory may accelerate functional recovery, rather than being merely a secondary outcome. From a treatment selection perspective, these results support the consideration of esketamine, particularly in individuals whose depressive episodes are characterized by marked psychomotor slowing, reduced cognitive energy, or difficulty sustaining attention, features often associated with poorer response to traditional monoaminergic antidepressants and with higher risk of functional nonremission. The absence of cognitive deterioration over long-term administration also supports the use of maintenance treatment when clinically indicated, especially in patients with recurrent or chronic symptom patterns.

Finally, these findings highlight the value of systematic cognitive assessment in clinical monitoring. Integrating brief, reliable cognitive measures (eg, DSST, TMT, RBANS indices, or digital processing speed tasks) into treatment follow-up may help identify patients who are benefiting cognitively from esketamine, as well as those who may require additional cognitive or rehabilitative interventions to optimize functional outcomes. This shifts the treatment target from symptom remission alone to a more comprehensive model of functional and cognitive recovery. Incorporating these observations into clinical decision-making suggests that esketamine may be particularly relevant for patients presenting with a cognitive-predominant depressive phenotype, characterized by slowed processing, attentional inefficiency, and reduced initiation capacity. Rather than applying a uniform treatment approach, identifying this profile at baseline may help clinicians select patients most likely to derive functional benefit from esketamine, while also clarifying when adjunctive cognitive interventions (eg, cognitive remediation therapy, occupational rehabilitation, structured goal-setting interventions) may be necessary. In this sense, cognition becomes not only a safety outcome, but a guide for treatment personalization: targeting patients whose recovery depends not only on mood improvement, but on the restoration of cognitive efficiency that supports agency, planning, and daily functioning.

### Limitations

Several limitations should be considered when interpreting these findings. First, the relatively small number of eligible studies represents an important limitation of this systematic review. Despite a comprehensive search strategy, only 6 studies met the predefined inclusion criteria, reflecting the current scarcity of longitudinal investigations specifically addressing cognitive outcomes associated with esketamine in TRD. Moreover, sample sizes were modest in several of the included cohorts, which constrains the precision and robustness of effect estimates, particularly for executive function outcomes. Consequently, the observed cognitive patterns should be interpreted as preliminary and hypothesis-generating rather than definitive.

Second, the cognitive assessment tools varied across studies, with different instruments used to evaluate similar domains, making direct quantitative comparison difficult. Relatedly, the timing of assessments was heterogeneous: some studies evaluated cognition within the early subacute phase (≤2 weeks), whereas others examined sustained (4 to 12 weeks) or long-term (≥3 to 10 months) cognitive effects; while this temporal variation was addressed through stratified interpretation, it limits the ability to infer continuous change over time.

Third, most included studies employed uncontrolled or open-label designs, lacking comparator groups. This substantially limits causal inference and reduces the certainty with which cognitive changes can be attributed specifically to esketamine rather than to nonspecific factors such as mood improvement, expectancy effects, practice effects, or concurrent treatments. In addition, adequately powered long-term controlled studies specifically designed to assess cognitive outcomes remain scarce, further limiting conclusions regarding sustained cognitive effects.

An additional limitation concerns the deliberate exclusion of studies investigating racemic ketamine. As outlined in the introduction, the present review focused exclusively on esketamine to ensure methodological coherence, given its distinct pharmacokinetic profile, standardized intranasal dosing, regulatory approval, and mandated clinical monitoring. Nevertheless, this choice necessarily limits the scope of the review, as evidence derived from racemic ketamine, despite pharmacodynamic overlap, was not considered. Consequently, the present findings should be interpreted within the specific context of esketamine treatment and may not be directly generalizable to other ketamine-based interventions.

Furthermore, the generalizability of the findings to older and clinically complex populations is limited. Older individuals were underrepresented across the included studies, and none were specifically designed to examine age-related vulnerability to cognitive effects. In addition, most studies, particularly registration trials, applied restrictive inclusion and exclusion criteria, often excluding patients with significant medical comorbidities, severe psychiatric comorbidities, or complex polypharmacy. As a result, the study samples may not fully reflect real-world patients with TRD, in whom age, multimorbidity, and cumulative treatment burden could modify cognitive outcomes. Moreover, the available literature focused primarily on core neurocognitive domains such as attention, memory, and executive functions, while other clinically relevant domains, including social cognition, emotion processing, and theory of mind, were not systematically assessed. Consequently, potential effects of esketamine on these aspects of cognition may have been missed.

Finally, publication bias cannot be excluded, as studies reporting cognitive decline or neutral outcomes may be less likely to be published. Taken together, these limitations underscore the need for larger, harmonized, prospective studies employing standardized cognitive batteries, appropriate control conditions, and consistent follow-up intervals to more precisely characterize the cognitive effects of esketamine in TRD.

## CONCLUSIONS

Current evidence, albeit limited, suggests that esketamine appears to be cognitively safe in adults with TRD, with no evidence of deterioration across attention, memory, or executive domains. Improvements in attention and processing speed were the most consistently observed findings, sometimes emerging before or in parallel with mood improvement, while memory and executive functions generally remained stable or improved over longer treatment durations. These cognitive effects may support functional recovery, particularly in individuals presenting with a cognitive-predominant depressive phenotype characterized by psychomotor slowing and attentional inefficiency. However, several of the reported cognitive improvements derive from open-label studies without control groups; therefore, the magnitude of these changes relative to treatment as usual or placebo cannot be definitively established. In addition, the overall quality of the available evidence remains constrained by the small number of studies, the predominance of uncontrolled designs, and the limited representation of older and clinically complex patients. Moreover, the long-term cognitive effects of sustained NMDA receptor antagonism remain insufficiently characterized. Future research with harmonized assessment protocols, larger real-world samples, and longer follow-up is needed to more precisely characterize the trajectory of cognitive change and to determine how cognitive outcomes may guide personalized treatment planning in TRD.

## Supplementary Material
