# Supplementary material for: Cognitive Effects of Esketamine in Treatment-Resistant Depression: A Systematic Review
Source: J Clin Psychopharmacol. 2026 Mar 24;46(5):612–24. doi: 10.1097/JCP.0000000000002165 (PMC13506185; doi:10.1097/JCP.0000000000002165)
Supplement: Supplementary file 1 [file jcp-46-612-s001.docx]

**Supplemental Digital Content**

**Search strings**

PubMed

(esketamine OR esketamine hydrochloride OR spravato) AND (cognition OR cognitive accessibility OR cognitive balance OR cognitive dissonance OR cognitive function OR cognitive structure OR cognitive symptoms OR cognitive task OR cognitive thinking)

PsychInfo

(esketamine OR spravato )

AND

(cognition OR cognitive OR accessibility OR cognitive OR balance OR cognitive OR dissonance OR {cognitive function} OR {cognitive structure} OR {cognitive symptoms} OR {cognitive task} OR {cognitive thinking})

Scopus

( TITLE-ABS-KEY ( esketamine OR spravato ) AND TITLE-ABS-KEY ( cognition OR cognitive OR accessibility OR cognitive OR balance OR cognitive OR dissonance OR {cognitive function} OR {cognitive structure} OR {cognitive symptoms} OR {cognitive task} OR {cognitive thinking})

**Table S1. Risk of bias: NIH Quality Assessment Tool for Before-After (Pre-Post) Studies With No Control Group**

**Criteria**

1. Was the study question or objective clearly stated?

2. Were eligibility/selection criteria for the study population prespecified and clearly described?

3. Were the participants in the study representative of those who would be eligible for the test/service/intervention in the general or clinical population of interest?

4. Were all eligible participants that met the prespecified entry criteria enrolled?

5. Was the sample size sufficiently large to provide confidence in the findings?

6. Was the test/service/intervention clearly described and delivered consistently across the study population?

7. Were the outcome measures prespecified, clearly defined, valid, reliable, and assessed consistently across all study participants?

8. Were the people assessing the outcomes blinded to the participants' exposures/interventions?

9. Was the loss to follow-up after baseline 20% or less? Were those lost to follow-up accounted for in the analysis?

10. Did the statistical methods examine changes in outcome measures from before to after the intervention? Were statistical tests done that provided p values for the pre-to-post changes?

11. Were outcome measures of interest taken multiple times before the intervention and multiple times after the intervention (i.e., did they use an interrupted time-series design)?

12. If the intervention was conducted at a group level (e.g., a whole hospital, a community, etc.) did the statistical analysis take into account the use of individual-level data to determine effects at the group level?

| ARTICLE | Q1 | Q2 | Q3 | Q4 | Q5 | Q6 | Q7 | Q8 | Q9 | Q10 | Q11 | Q12 | R1 | R2 | Final |
| --- | --- | --- | --- | --- | --- | --- | --- | --- | --- | --- | --- | --- | --- | --- | --- |
| **(26)** | Y | Y | Y | N | Y | Y | Y | N | N | N | N | NA | Fair | FAIR | Fair |
|  | Y | Y | Y | N | Y | Y | Y | N | N | N | N | NA |  |  |  |
| **(29)** | Y | Y | Y | NR | N | Y | Y | N | N | Y | N | NA | Good | GOOD | Good |
|  | Y | Y | N | NR | N | Y | Y | N | Y | Y | Y | NA |  |  |  |
| **(28)** | Y | Y | Y | CD | N | Y | Y | N | N | Y | N | NA | Good | GOOD | Good |
|  | Y | Y | Y | NR | Y | Y | Y | N | N | Y | Y | NA |  |  |  |
| **(17)** | Y | Y | Y | NR | N | Y | Y | N | CD | Y | Y | NA | Fair | FAIR | fair |
|  | Y | Y | Y | Y | N | Y | Y | N | NA | Y | Y | NA |  |  |  |

*CD, cannot determine; NA, not applicable; NR, not reported
**Table S2**. **Risk of bias: NIH Quality Assessment Tool for Case Series Studies**

**Criteria**

1. Was the study question or objective clearly stated?

2. Was the study population clearly and fully described, including a case definition?

3. Were the cases consecutive?

4. Were the subjects comparable?

5. Was the intervention clearly described?

6. Were the outcome measures clearly defined, valid, reliable, and implemented consistently across all study participants?

7. Was the length of follow-up adequate?

8. Were the statistical methods well-described?

9. Were the results well-described?

| ARTICLE | Q1 | Q2 | Q3 | Q4 | Q5 | Q6 | Q7 | Q8 | Q9 | R1 | R2 | Final |
| --- | --- | --- | --- | --- | --- | --- | --- | --- | --- | --- | --- | --- |
| **(27)** | Y | Y | NA | Y | Y | Y | N | N | Y | Fair | FAIR | fair |
|  | Y | Y | NA | Y | Y | Y | N | N | Y |  |  |  |

*CD, cannot determine; NA, not applicable; NR, not reported

**Table S3. Risk of bias: NIH Quality Assessment Tool for Systematic Reviews and Meta-Analyses**

**Criteria**

1. Is the review based on a focused question that is adequately formulated and described?

2. Were eligibility criteria for included and excluded studies predefined and specified?

3. Did the literature search strategy use a comprehensive, systematic approach?

4. Were titles, abstracts, and full-text articles dually and independently reviewed for inclusion and exclusion to minimize bias?

5. Was the quality of each included study rated independently by two or more reviewers using a standard method to appraise its internal validity?

6. Were the included studies listed along with important characteristics and results of each study?

7. Was publication bias assessed?

8. Was heterogeneity assessed? (This question applies only to meta-analyses.)

| ARTICLE | Q1 | Q2 | Q3 | Q4 | Q5 | Q6 | Q7 | Q8 | R1 | R2 | Final |
| --- | --- | --- | --- | --- | --- | --- | --- | --- | --- | --- | --- |
| **(25)** | Y | Y | CD | NR | CD | Y | N | NA | Fair | FAIR | fair |
|  | Y | Y | NA | NA | CD | Y | N | Y |  |  |  |

*CD, cannot determine; NA, not applicable; NR, not reported
